# Varicose Ulcers of the Legs

**Published:** 1898-01

**Authors:** 


### VARICOSE ULCERS OF THE LEGS.

Simonelli (*Revue Medicale*, Oct. 6, 1897) recommends a powder composed of fifty parts of chloride of sodium and five parts of pulverized menthol. Both substances should be in the form of an impalpable powder, and intimately admixed.

